# A longitudinal study on parotid and submandibular gland changes assessed by magnetic resonance imaging and ultrasonography in post-radiotherapy nasopharyngeal cancer patients

**DOI:** 10.1259/bjro.20200003

**Published:** 2020-09-02

**Authors:** Vincent W.C. Wu, Michael TC Ying, Dora LW Kwong, Pek-Lan Khong, Gary KW Wong, Shing-yau Tam

**Affiliations:** 1 Department of Health Technology and Informatics, Hong Kong Polytechnic University, Hung Hom, Kowloon, Hong Kong; 2 Department of Clinical Oncology, Li Ka Shing Faculty of Medicine, The University of Hong Kong, Pok Fu Lam, Hong Kong; 3 Department of Radiology, Li Ka Shing Faculty of Medicine, The University of Hong Kong, Pok Fu Lam, Hong Kong; 4 Department of Clinical Oncology, Queen Mary Hospital, Sha Tin, Hong Kong

## Abstract

**Objectives:**

With regard to the intensity modulated radiotherapy (IMRT) of nasopharyngeal carcinoma (NPC) patients, this longitudinal study evaluated the radiation-induced changes in the parotid and submandibular glands in terms of gland size, echogenicity and haemodynamic parameters.

**Methods:**

21 NPC patients treated by IMRT underwent MRI and ultrasound scans before radiotherapy, and at 6, 12, 18 and 24 months after treatment. Parotid and submandibular gland volumes were measured from the MRI images, whereas the parotid echogenicity and haemodynamic parameters including the resistive index, pulsatility index, peak systolic velocity and end diastolic velocity were evaluated by ultrasonography. Trend lines were plotted to show the pattern of changes. The correlations of gland doses and the post-RT changes were also studied.

**Results:**

The volume of the parotid and submandibular glands demonstrated a significant drop from pre-RT to 6 months post-RT. The parotid gland changed from hyperechoic before RT to either isoechoic or hypoechoic after treatment. The resistive index and pulsatility index decreased from pre-RT to 6 month post-RT, then started to increase at 12 month time interval. Both peak systolic velocity and end diastolic velocity increased after 6 months post-RT then followed a decreasing trend up to 24 months post-RT. There was mild correlation between post-RT gland dose and gland volume, but not with haemodynamic changes.

**Conclusions:**

Radiation from IMRT caused shrinkage of parotid and submandibular glands in NPC patients. It also changed the echogenicity and vascular condition of the parotid gland. The most significant changes were observed at 6 months after radiotherapy.

**Advances in knowledge:**

It is the first paper that reports on the longitudinal changes of salivary gland volume, echogenicity and haemodynamic parameters altogether in NPC patients after radiotherapy. The results are useful for the prediction of glandular changes that is associated with xerostomia, which help to provide timely management of the complication when the patients attend follow-up visits.

## Introduction

Xerostomia is a common radiation-induced complication in post-radiotherapy (RT) nasopharyngeal carcinoma (NPC) patients.^1^ This complication affects mastication and swallowing and increases susceptibility to oral infections and dental problems, which subsequently degrade the quality of life in post-RT NPC patients.^2,3^ In a radical course of radiotherapy to NPC patients, portions of major salivary glands including the parotid gland and submandibular gland are often irradiated to high dose. It has been demonstrated that xerostomia was dependent on the radiation dose delivered to the salivary glands in NPC patients.^4^ Its incidence varied greatly from 39.3 to 82.1% depending on the RT techniques.^5,6^ Although the recent introduction of intensity modulated radiotherapy (IMRT) can achieve better sparing of parotid and submandibular glands,^7–10^ the irradiation of these glands is still inevitable and xerostomia has been frequently reported.^11^ Furthermore, radiotherapy of NPC often delivered with concurrent chemotherapy, bodyweight loss, primary tumour shrinkage and parotid gland shrinkage during the relatively long (over 7 weeks) IMRT course are common. These changes could cause the medial migration of the parotid gland, which subsequently increase the parotid dose and thereby the chance of developing xerostomia.^12,13^ It was reported in an animal study that portion of parotid and submandibular gland that received 70 Gy were found to have significant fibrosis, acinar atrophy and parenchymal loss.^14^ However, at present, the detail mechanism of radiation-induced xerostomia is still largely unknown.

The onset of xerostomia was proposed to be due to the damages to the signal transduction system plasma membrane of acinar cells in early stage. The subsequent result of xerostomia and its recovery was associated with the damages of salivary gland stem cells that reduce cell renewal ability in later stage.^2,15^ (Salivary gland recovery is usually referred to the increase of saliva flow after it has reached the minimum). Due to the heterogeneous nature of the salivary gland structure, the stem cells in parotid glands are mainly located at the anterolateral segment. It also reported that the superior portions of the parotid gland were the most influential region for xerostomia recovery.^16^ It was expected that by keeping the mean dose of less than 25 Gy at these regions of the gland, the function of the post-RT parotid gland could be better preserved.^17^

Post-RT ultrasound examination of salivary glands demonstrated changes in echotexture from a homogenous speckle pattern in normal condition to a heterogeneous structure, which could be hypo- or isoechoic to adjacent muscles with multiple hyperechoic spots.^18,19^ It has been demonstrated that induced salivary gland injury was associated with ultrasound echogenicity and it could be effectively assessed by echo histograms.^20^ Furthermore, the changes in vascular condition including haemodynamic parameters such as the resistive index (RI) and flow velocity of the post-RT parotid gland using Doppler ultrasonography have also been reported in which normal gland demonstrated higher RIs than post-RT glands.^21^ Despite some studies have reported that there was recovery of the salivary gland after radiotherapy, it was not a complete restoration of the normal saliva production and the post-RT impact on the glands was longlasting.^22^ A more comprehensive understanding of the progression of the morphology and haemodynamic changes with respect to time is important to predict the development of xerostomia and provide better management for the patients.

MRI has excellent spatial resolution and is superior to CT in delineating soft tissue structures and radiation free. Ultrasonography can provide supplementary information apart from MRI such as the texture of the gland by measuring the echogenicity, and haemodynamic information including the peak systolic velocity (PSV) and RI using the Doppler ultrasound.^21^ Since these two modalities do not involve radiation, they can be performed on more regular basis as applied in this longitudinal study. The objectives of this study are to assess the post-RT changes of volume of the parotid and submandibular glands, the echogenicity and haemodynamic of parotid gland with respect to time and evaluate their correlations with the radiation dose received in NPC patients treated with IMRT.

## Methods and materials

21 NPC patients (age range: 29–62, median age: 51) treated with IMRT between April to Dec 2017 were recruited. The patient characteristics are shown in Table 1. Written informed consent was obtained from the patients to join the study before the start of the treatment. Ethics approval was obtained from the Research Ethics Committee of the Hong Kong Polytechnic University and from Institutional Research Board of the University of Hong Kong. Each patient underwent planning CT of the head and neck region covering the whole skull down to the level of supra sternal notch. The CT data were transferred to radiotherapy treatment planning system (Eclipse ^TM^, Varian Medical Systems, Palo Alto, CA) where the IMRT plans were generated. The routine IMRT plan consisted of nine equally spaced beams covering the base of skull down to the lower neck using 6 MV photon. The planning target volumes (PTVs) of the nasopharynx and neck lymphatics were prescribed with 70 and 66 Gy respectively. Since the parotid and submandibular glands are the major salivary glands that produce over 80% of saliva in human,^23^ only these two glands were included in this study. Dose parameters of parotid and submandibular glands including maximum dose (D_max_), minimum dose (D_min_) and mean dose (D_mean_) were obtained from their respective dose–volume histograms (DVH) generated from the treatment planning system.

**Table 1.** Patient characteristics (*n* = 21)

	Number of patients (%)
Gender	
Male	13 (61.9%)
Female	8 (38.1%)
Tumour Stage (AJCC)	
I	2 (9.5%)
II	4 (19.0%)
III	7 (33.3%)
IV	6 (28.6%)
Unknown	2 (9.5%)
Chemotherapy	
Yes	16 (76.2%)
No	3 (14.3%)
Unknown	2 (9.5%)

AJCC: American Joint Committee on Cancer

The assessment of the volume changes of the parotid and submandibular glands were performed using MRI. Each patient underwent MRI scans before the start of radiotherapy (pre-RT) and at 6, 12, 18 and 24 months post-RT. During the scan, the patient lied supine on the examination couch with the head straight. For the scanning of parotid gland, multicoil head coil was used. The head was adjusted so that the interpupillary line was parallel to the couch. For the submandibular gland, the anterior neck coil was used with the patient positioned so that the longitudinal alignment light lied in the midline and the horizontal alignment passed through the angle of jaw. The scanning volume covered from the base of skull to the hyoid bone. The scanning sequences included: T1 axial and sagittal, and T2 axial and sagittal scans with slice thickness of 3 mm and no inter slice gap. Images generated from the scanner were transferred to the workstation equipped with MIM Maestro (MIM Software Inc, Cleveland, OH) where the images were displayed and the delineation of the parotid and submandibular glands were conducted. The volume of each gland was calculated by the system.

For each patient, ultrasound scan of the parotid gland was performed at similar time intervals as the MRI (*i.e.* pre-RT, 6, 12, 18 and 24 months post-RT). Apart from assessing the tissue echogenicity of the parotid gland, the haemodynamic parameters including vascular resistance [RI and pulsatility index (PI)] and blood flow velocity parameters [peak systolic velocity (PSV) and end diastolic velocity (EDV)] were measured using the power Doppler and spectral Doppler ultrasound. RI and PI indicated the pressure exerted on the blood vessels while PSV and EDV indicated blood flow velocity during the systole and diastole phases of the cardiac cycle respectively. Ultrasound examination was conducted using a 12 to 5 MHz linear transducer (Philips HDI 5000, Bothell, WA). Prior to the start of ultrasound examination, the patient lied supine on the examination couch for at least 5 min to ensure accurate measurement of resting echogenicity and blood flow. Greyscale ultrasonography was conducted for accessing echogenicity of parotid glands and the comparison of echogenicity was made with respect to the adjacent masseter muscle as hyper-, iso- or hypoechoic. The use of masseter muscle as the reference has been reported by previous studies^21,24^ with the rationale that the muscle was situated at the low dose region (<20 Gy), and therefore was not expected to have morphological changes due to radiation. In addition, this could allow convenient and efficient echogenicity assessment within the same image. High sensitivity was utilised in the power Doppler ultrasound settings with a low wall filter to allow detection of low blood flow. Pulsed repetition frequency (PRF) was 700 Hz with medium persistence used. For spectral Doppler ultrasound, the sample volume was standardised for 1 mm with a low wall filter. The PRF was adjusted until the spectral waveforms were demonstrated without aliasing. Angle correction was 60° or below. The haemodynamic measurements were evaluated at random locations within three vessels that consistently demonstrated three consecutive Doppler spectral waveforms and mean values of RI, PI, PSV and EDV were calculated. Trend lines were plotted to assess the percentage post-RT changes of parotid and submandibular gland volume, and parotid haemodynamic parameters. The difference of percentage changes of gland volume and haemodynamic parameters over time were assessed by repeated measures ANOVA. Also, the correlation between these values and dose parameters were analysed using the Pearson correlation test. The level of significance of the difference in echogenicity was calculated by McNemar test. Statistical Product and Service Solutions (SPSS) (v. 22) was employed for statistical analyses.

## Results

**Figure 1.**
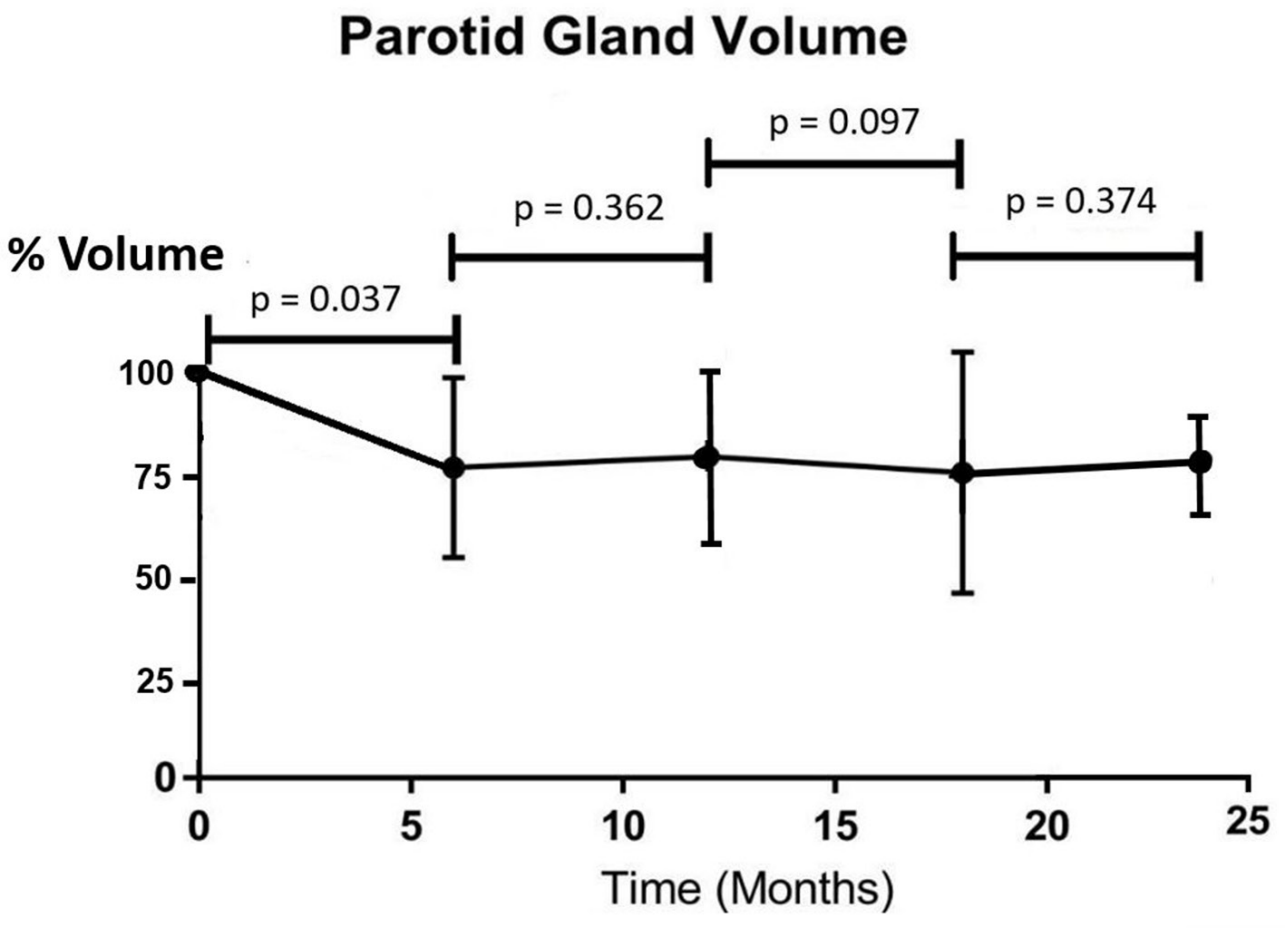
Trend line showing the percentage change of mean parotid gland volume after radiotherapy (*n* = 42).

**Figure 2.**
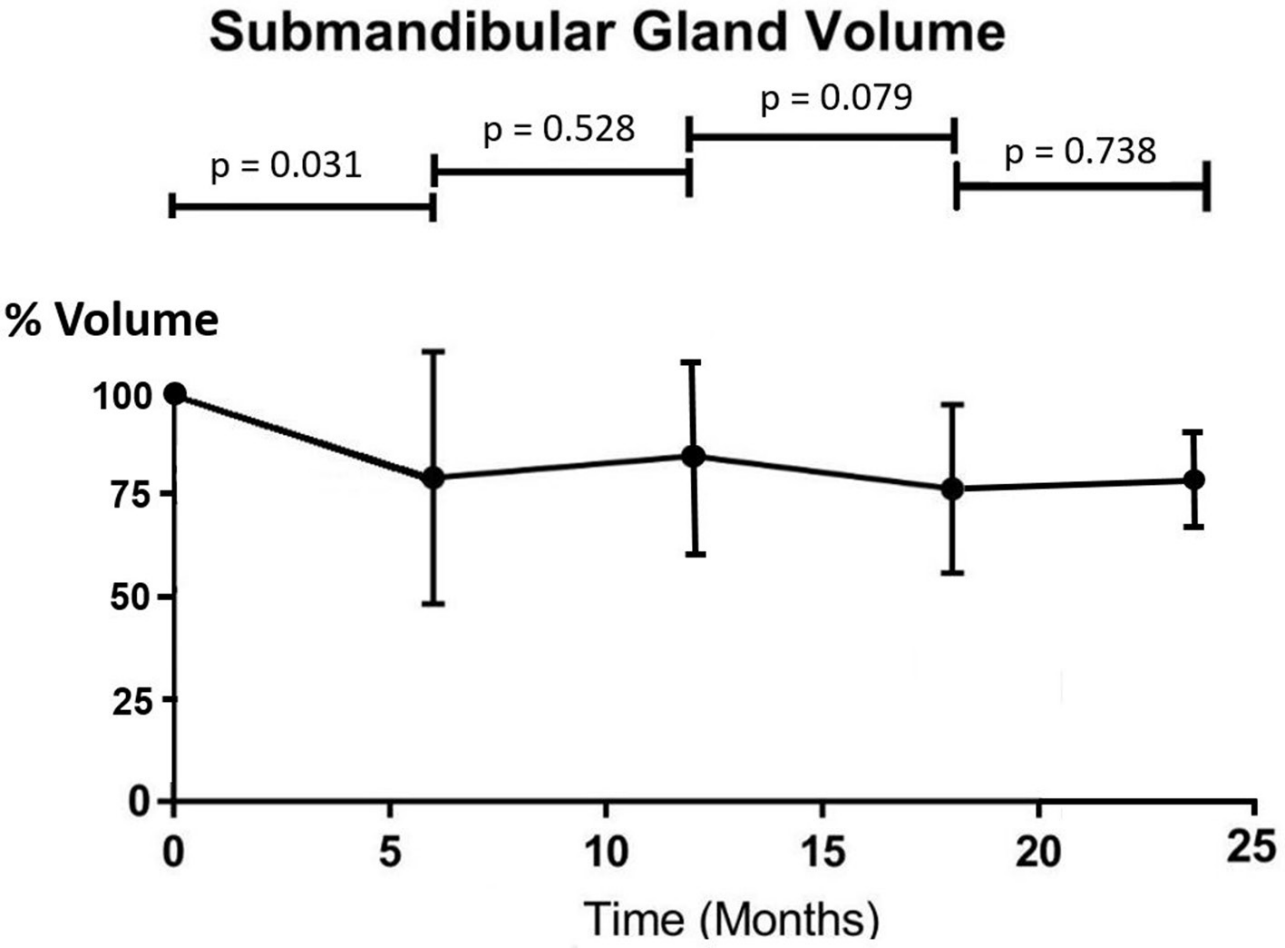
Trend line showing percentage change of mean submandibular gland volume after radiotherapy (*n* = 42).

All patients completed the IMRT treatment uneventfully. Similar doses were received by the parotid gland and submandibular gland, with the maximum dose over 70 Gy and mean dose around 37 Gy (Table 2). The percentage volume of the parotid gland demonstrated a significant drop from pre-RT to 6 months post-RT (*p* = 0.037) and became fairly stable in the subsequent time intervals (*p* > 0.05 between two consecutive intervals) (Figure 1). The overall mean volume reduction was 2.9 ± 4.0 cm^3^, which was 25.8% of the pre-RT parotid gland volume. The submandibular gland followed similar trend as the parotid gland. Its volume showed a significant drop at 6 months after radiotherapy (*p* = 0.031) and demonstrated no significant differences in the following time intervals (*p* > 0.05) (Figure 2). The overall mean volume reduction was 1.7 ± 1.0 cm^3^, which was 21.8% of the pre-RT submandibular gland volume. Moreover, there were mild correlations observed between post-RT gland volume changes with their mean doses received. (Pearson correlation test, *p* = 0.044 and *p* = 0.050 respectively).

**Table 2.** Parotid and submandibular dose parameters (*n* = 42)

	Mean ± SD
Parotid gland	
D_max_	70.9 ± 1.3 Gy
D_min_	12.7 ± 2.1 Gy
D_mean_	38.0 ± 4.5 Gy
Submandibular gland	
D_max_	71.0 ± 1.6 Gy
D_min_	13.3 ± 3.1 Gy
D_mean_	36.9 ± 3.5 Gy

D_max_: Maximum dose; D_min_: Minimum dose; D_mea_: Mean dose; SD: Standard deviation

**Figure 3.**
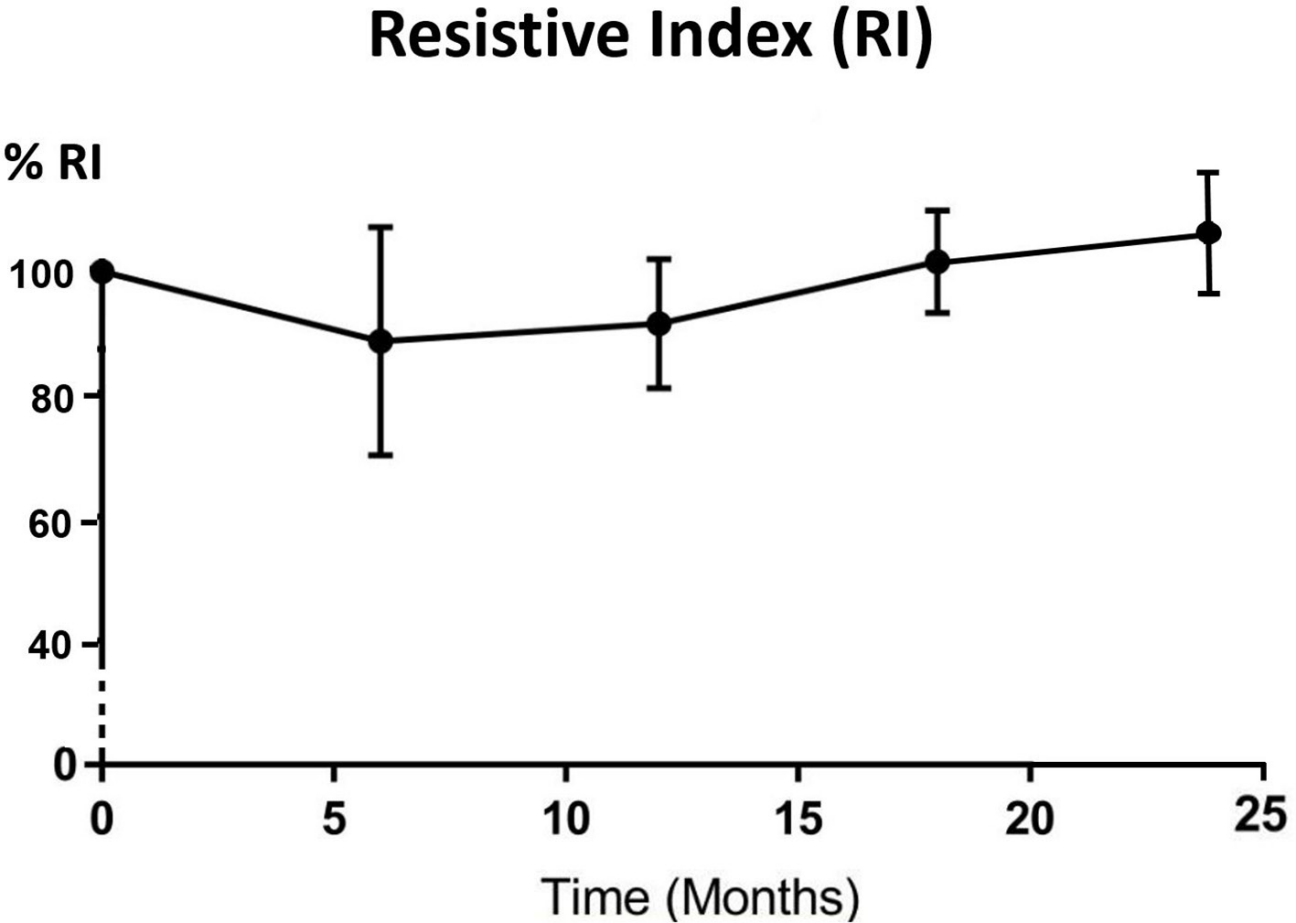
Trend line showing percentage change of mean RI after radiotherapy (*n* = 42). RI, resistive index.

**Figure 4.**
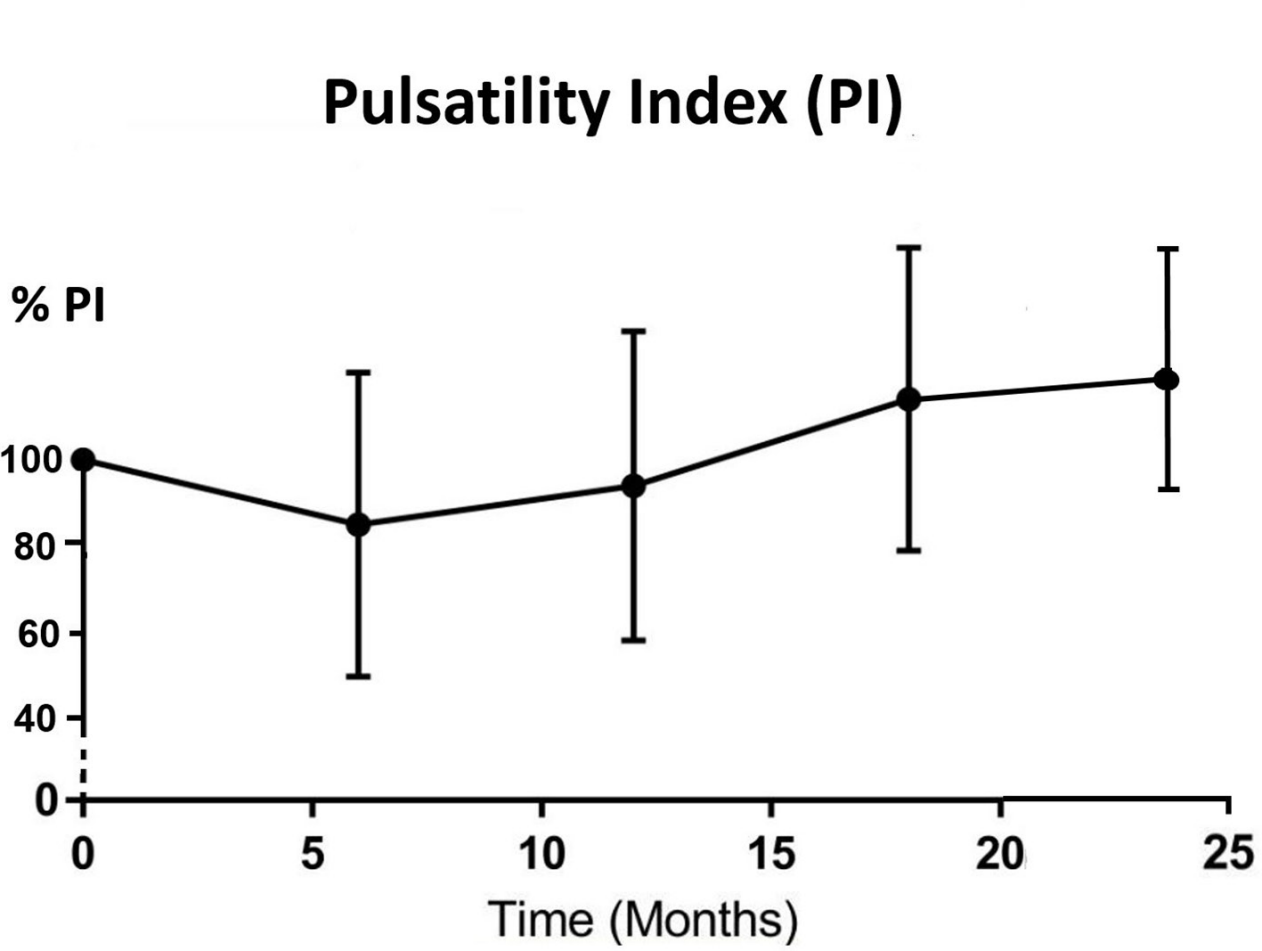
Trend line showing percentage change of mean PI after radiotherapy (*n* = 42). PI, pulsatility index.

**Figure 5.**
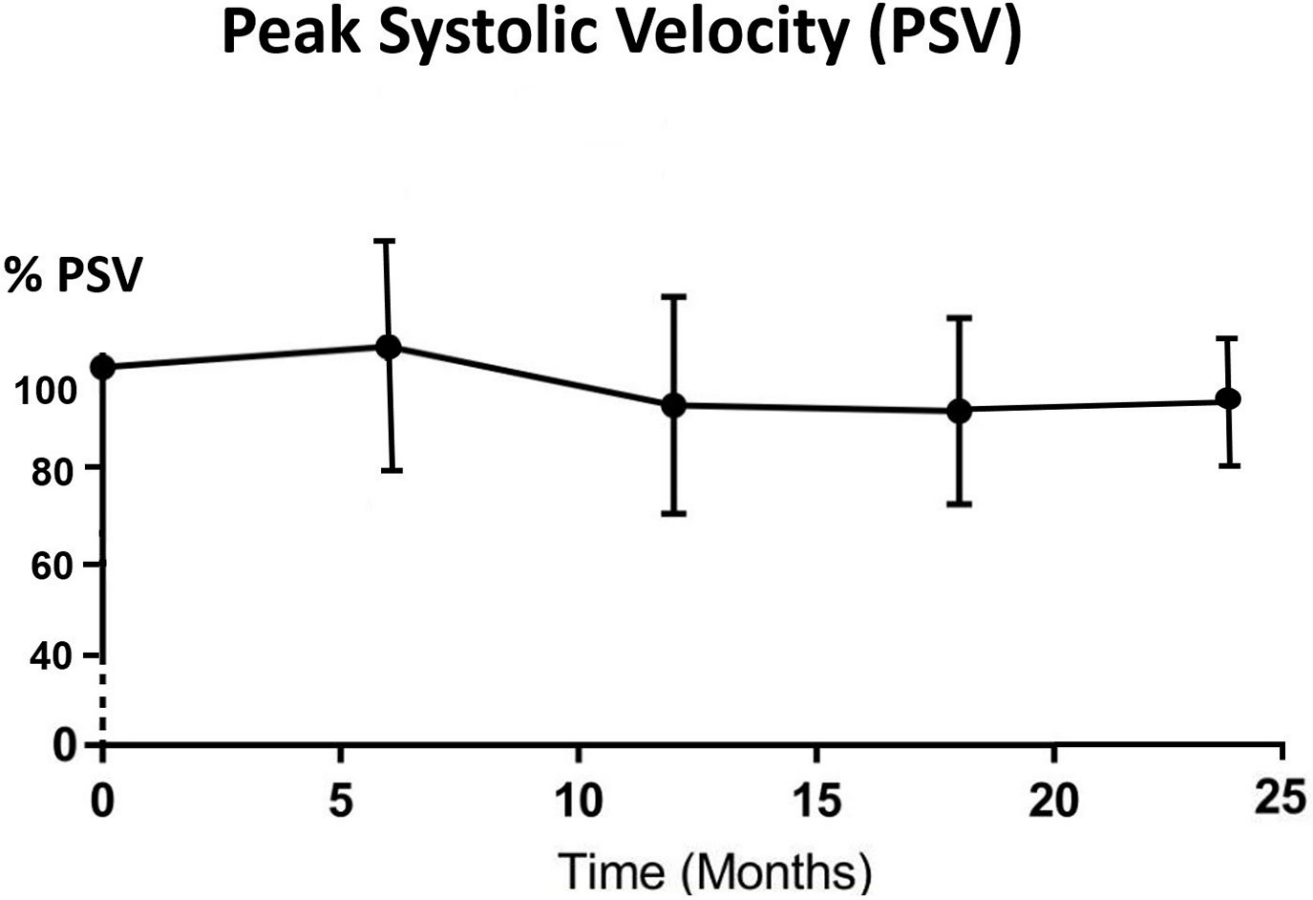
Trend line showing percentage change of mean PSV after radiotherapy (*n* = 42). PSV, peak systolic velocity.

**Figure 6.**
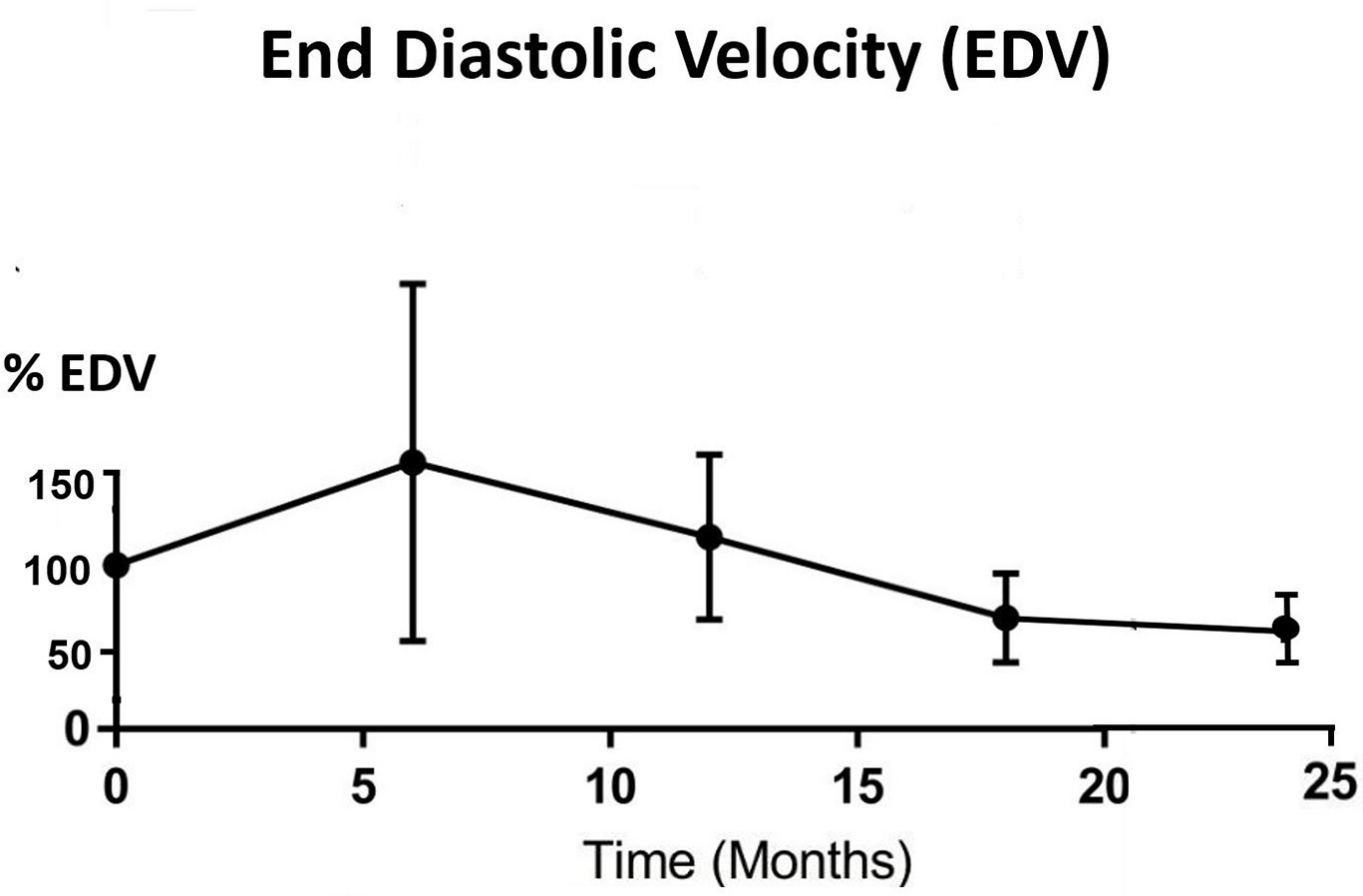
Trend line showing percentage change of mean EDV after radiotherapy (*n* = 42). EDV, end diastolic velocity.

All parotid glands were hyperechoic in comparison with adjacent muscle before IMRT treatment. The echogenicity changed to either hypoechoic (33.3%–60.0%) or isoechoic (40%–66.7%) after 6 months post-RT (Table 3). Significant differences (*p* < 0.05) were found between pre-RT and all post-RT time intervals while no significant difference was found between different post-RT time intervals. For the haemodynamic study of the parotid gland, RI and PI decreased from pre-RT to 6 month post-RT, then started to increase in 12 month time interval and continued to 24 month post-RT, where the reading was slightly higher than that of pre-RT (Figures 3 and 4). Both PSV and EDV increased after 6 months post-RT, then followed a decreasing trend up to 24 month post-RT (Figures 5 and 6). Similar to the percentage gland volume change, there was no significant correlation between post-RT parotid haemodynamic changes and doses received by the gland (*p* > 0.05).

**Table 3.** Echogenicity study of pre- and post-RT parotid glands

	Number of parotid glands (%)				
	Pre-RT	6 months post-RT	12 months post-RT	18 months post-RT	24 months post-RT
Hyperechoic	42 (100%)	0 (0%)	0 (0%)	0 (0%)	0 (0%)
Isoechoic	0 (0%)	26 (61.9%)	28 (66.7%)	23 (55.0%)	17 (40%)
Hypoechoic	0 (0%)	16 (38.1%)	14 (33.3%)	19 (45.0%)	25 (60%)

RT: Radiotherapy

## Discussion

Our study demonstrated that radiation therapy to NPC patients led to shrinkage of the parotid and submandibular glands, which was more prominent during the first 6 months after treatment. The percentage volume loss in parotid gland than the submandibular gland (25.8% *vs* 21.8%) in this study was in line with the result reported by Wang et al.^25^ They reported that parotid glands received similar mean dose as submandibular glands but experienced greater volume loss. It has been reported that shrinkage of salivary gland during IMRT was associated with the reduction of salivary flow rate and subsequently the severity of xerostomia.^26^ After the 6 month interval, both gland volumes remained fairly constant with no significant increase or decrease during the study period. When correlating the current results with that of another study by our team,^27^ it was found that there was relationship between the radiological (MRI) findings and the clinical outcomes including saliva flow rate and severity of xerostomia in post-RT patients. The saliva flow rates also demonstrated significant percentage reduction at 6 months post-RT when the dryness of mouth was most severe. In line with MRI findings, these clinical parameters became fairly stable in subsequent time intervals after 6 months. In addition, since our study showed that there was correlation between glands size changes and gland doses, higher radiation dose to the salivary glands would cause greater volume reduction. This echoed our previous study reporting that gland doses could be used to predict gland volume change.^22^ Although this present study did not assess the gland size during radiotherapy, such shrinkage was started around the middle and late stage of the treatment course according to previous studies.^28,29^ Fung et al^30^ reported that parotid glands shrank at a mean rate of 1.35% per day and demonstrated an average medial migration of 0.34 cm after a course IMRT in NPC patients. Since changes in location and size during radiotherapy course would lead to increase dose to the salivary glands, adaptive radiotherapy with re-planning around mid- and/or late course of treatment were suggested.^28,29,31,32^

Salivary glands have been demonstrated to show recovery after the completion of radiotherapy.^33,34^ Van Luijk et al suggested that the stem or progenitor cells in the human parotid gland, which provided the regenerative capacity of the gland tissue in the irradiated region, were responsible for the recovery of parotid gland.^15^ A study by Sim et al^11^ reported that both parotid and submandibular glands demonstrated volume recovery after 2 years post-RT, whereas our study demonstrated that the gland volumes tended to remain stable at 24 months. When comparing to our previous study on NPC patients treated by conventional radiotherapy (ConRT),^22^ our current study on IMRT patients experienced less parotid gland volume reduction (18.8% in IMRT versus 35% in ConRT). This echoed the fact that IMRT has the advantage of better sparing the parotid gland and less post-RT shrinkage compared to conventional RT.^6^

In this study, all parotid glands were hyperechoic before IMRT but changed to iso- or hypoechoic after treatment, with the isoechoic status constituting higher percentages in all the three post-RT time intervals. Normal parotid glands were hyperechoic because before irradiation, the densely packed serous secretory acini and translucent secretory granules could act as reflective interface, and together with the fatty infiltration constituted the relatively hyperechoic echo-pattern.^14,35^ This result was in line with a cross-sectional study by Ying et al.^21^ The reduced echogenicity in post-RT parotid glands could be due to the diffuse infiltration of lymphocytes, vacuolated acinar cell cytoplasm and loss of secretory granules leading to poorer cell compactness.^14^ Such changes involving the reduction of acinar cells in the parotid gland could be associated with the reduction of saliva production leading to xerostomia. In our study, none of the parotid gland returned to the hyerechoic status within the 24 months period after RT indicating that such changes were not reversible in such time frame. Furthermore, our study showed that the change of echogenicity occurred at the 6 month post-RT time interval and continued throughout the study period, which echoed the results reported by Imanimoghaddam et al.^19^

Our current study is the first longitudinal study that has monitored the haemodynamic changes of parotid gland in post-RT NPC patients. For RI and PI, the indexes dropped during the first 6 months of post-RT period and gradually recovered afterwards. The vascular resistance changes between pre-RT and 6 months post-RT could be caused by the lowered compression pressure due to the reduced number of secretory acini and granules,^22^ which was also the reason for the reduction of gland volume at 6 months post-RT. As the reduction became stabilised after 6 months, the vascular resistance then gradually built up and therefore followed an increasing trend. The pattern of PSV and EDV changes was roughly the opposite of the vascular resistance (PI and RI). They both demonstrated increase in the first 6 months after treatment followed by a decreasing trend. It was logical to see the increase in vascular velocity increase as the resistance decreased. In addition, the initial increasing trend might also be due to the inflammatory changes and recovery action from radiation-induced microvascular damage during the first 6 months.^36^ While for the monitoring period after 6 months, the post-RT fibrosis of blood vessels might lead to the recovery of vascular resistance indexes; and the less organised vascular architecture by acinar atrophy and parenchymal loss contributed to the reduced blood velocity.^14,23,37^ Based on these results, the 6 month post-RT intervals appeared to be the turning point of the volume and haemodynamic parameters changes in the parotid gland. Therefore, patient follow-up during the first 6 months after RT is important to monitor the condition of xerostomia so as to provide prompt patient care. The current study demonstrated that changes in haemodynamic parameters were not dependent on the radiation dose received by the parotid gland, which was in line with our previous cross-sectional study.^22^ It can therefore be speculated that with mean dose of over 35 Gy to the salivary gland (as recorded in this study), similar pattern of vascular changes would be expected in the post-RT NPC patients regardless of the exact absolute dose.

Furthermore, it is worth mentioning that recently development of radiomics has been used to predict radiation-induced xerostimia. Examples of these include parotid gland fat-related MRI biomarkers,^38^ dosiomic and demographic features,^39^ F-FDG positron emission tomography image biomarkers^40^ and CTand MR radiomics.^41^ Such technology will also facilitate the prompt management of xerostomia in patients after radiotherapy.

## Conclusions

With regard to IMRT of NPC patients, radiation caused shrinkage of the parotid and submandibular glands. The most significant volume reduction took place at 6 month post-RT. All parotid glands were hyperechoic before radiotherapy but changed to either iso- or hypoechoic after the completion of treatment. In terms of haemodynamic status, parotid glands demonstrated increased in vascular resistance (PI and RI) in the first 6 months and started to decrease afterward, whereas the vascular velocity (PSV and EDV) showed opposite trends relative to the vascular resistance. There were mild correlations between the mean gland dose and the changes in gland size, but not with the haemodynamic parameters.
